# Supplementary material for: Anterior Colporrhaphy and Paravaginal Repair for Anterior Compartment Prolapse: A Review
Source: Medicina (Kaunas). 2024 Nov 14;60(11):1865. doi: 10.3390/medicina60111865 (PMC11596843; doi:10.3390/medicina60111865)
Supplement: Supplementary file 1 [file medicina-60-01865-s001.zip › medicina-3239517-supplementary.pdf]

Supplement S1. Search strategies in PubMed database (from 1964-2024 Aug 31)

**Items**	**Specification**
Timeframe	1964 to 2024 Aug 31
Database	PubMed
Search terms used	“endometrial atypical hyperplasia”, “endometrial cancer”
Inclusion and exclusion criteria	All references were SCI-indexed articles The language is English
Selection process	Two independent reviewers evaluated the titles and abstracts to determine eligibility.
